# Assessment of Metastatic Colorectal Cancer (CRC) Tissues for Interpreting Genetic Data in Forensic Science by Applying 16 STR Loci among Saudi Patients

**DOI:** 10.31557/APJCP.2021.22.9.2797

**Published:** 2021-09

**Authors:** Wedad Saeed Al-Qahtani, Tahani Mohamed Al-Hazani, Fatmah Ahmed Safhi, Mashael Alhumaidi Alotaibi, Dalia Mostafa Domiaty, Salha M. S. Al-Shamrani, Eman Alshehri, Amani Mohammed Alotaibi, Saad Alkahtani

**Affiliations:** 1 *Department of Forensic Sciences, College of Criminal Justice, Naif Arab University for Security Sciences, Riyadh 11452, Saudi Arabia. *; 2 *Department of Biology, College of Sciences and Humanities, Prince Sattam Bin Abdulaziz University, Al-Kharj, 11940, Saudi Arabia. *; 3 *Department of Biology, College of Science, Princess Nourah bint Abdulrahman University, Riyadh, Saudi Arabia.*; 4 *Department of Biology, College of Science, Jouf University, Saudi Arabia. *; 5 *University of Jeddah, College of Science, Department of Biology, Jeddah 21493, Saudi Arabia. *; 6 *Department of Zoology, College of Science, King Saud University, Saudi Arabia. *; 7 *King Saud Medical City, Riyadh, Saudi Arabia. *

**Keywords:** Neoplastic tissues, Colorectal cancer tissues (CRC), adjoining non-cancerous tissue marginals (N-CRC)

## Abstract

**Background:**

In forensic science, there are cases when the only available provider of biological data is samples of malignant tissues. It can be useful in identification and/or paternity tests. Still, such samples have ambiguities because of microsatellite instability (MSI) and loss of heterozygosity (LOH) effects, being often related to neoplasia.

**Methods:**

This research evaluates 16 autosomal short tandem repeat (STR) loci (traditional in forensic investigations) to get genetic data. MSI and LOH were estimated in DNA patterns derived from 73 Saudi respondents (30 healthy individuals and 43 persons with diagnosed colorectal cancer (CRC). Upon deriving DNA from blood, CRC specimens were obtained in both groups, along with the adjoining normal non-cancerous tissues (N-CRC). All specimens and 16 loci (15 STR loci and Amelogenin) were evaluated. Moreover, both colorectal samples were histologically analyzed utilizing HandE staining.

**Results:**

Findings revealed non-essential variability in genetic information because of MSI and/or LOH. In CRC, mutations rates were 0.42% (MSI) and 1.62% (LOH). In N-CRC, mutation rates were 0.00% (MSI) and 0.59% (LOH). Further, LOH-related deviations were recorded in 5 loci out of 16. MSI-related deviations were recorded in 4 out of 16 loci, being present in CRC samples only. Genetic deviations within the marker loci might inform about false homozygosity/heterozygosity. Similarly, false gender might come from improper interpretation of DNA profiles. Finally, histopathological trials showed considerable histopathological alterations contrasted to N-CRC.

**Conclusion:**

This study is unique in demonstrating the application of 16 autosomal STRs from CRC samples and their comparison with the adjoining N-CRCs in Saudi participants, contributing to the field of forensic science. The experiment revealed no considerable distinctions, while showing that cancer tissues might display MSI and LOH effects that might challenge data interpretation, if STRs are to be applied in the forensic investigation.

## Introduction

Colorectal cancer (CRC) is viewed as a prevalent malignant formation developed in the gastrointestinal tract. According to world statistics, it is the third most prevalent type of cancer and the fourth most dominant factor of lethal outcome globally (Chen et al., 2014). Life risk percentage in regions of West Europe and North America among local populations is equal to almost 5% (Lichtenstein et al., 2000). From etiology perspective, the illness can be triggered by genetic/inherited and environmental factors, although 1/3 of disease dispersion relates to inborn biological variables (Lichtenstein et al., 2000).

It is known that microsatellite instability (MSI) by short tandem repeats (STRs) become valuable providers of information for examining genetic diseases and genetic oncology. In terms of forensics, they can be utilized for paternity tests and identity establishing cases; moreover, instruments are applied for linkage disequilibrium evaluation as well as genome mapping. Other studies have indicated (Dang et al., 2020) that the valuable input secured by these polymorphisms is stipulated by variability quality. Still, stability of data is a main term of receiving valid outcomes, which is why it all depends on scientific insight about mutation rates and origins of mutational patterns. MSI provides a context where an allele in the germline microsatellite has actually acquired or deprived certain repeat units; in other words, it has come through a somatic alteration in terms length. As a rule, extended MSI informs about the so-called mismatch repair deficiency (MMRD), which might be a source of many mutations in cancer-associated genes, provoking carcinogenesis process and tumor development. In this sense, MSI is often identifiable in selected types of cancer, and CRC in not an exclusion (Lynch HT and De la Chapelle A, 2003).

The effect of loss of heterozygosity (LOH) represents a case when the whole gene disappears. In this event, LOH is presented with copy number losses (a type titled CNL-LOH) as well as copy number neutral LOH (a type titled CNN-LOH). In case of CNL-LOH, the entire chromosome or only its part disappears. In case of CNN-LOH, disappearance takes place either due to a homologous recombination behavior (“gene 35 conversion”) or due to the fact that the engaged chromosome was copied (prior to or post the LOH case) (Ryland et al., 2015). LOH effect is heavily attributed to the removal of an allele of the wild type in people with a syndrome of high cancer hereditary potential. Additionally, this involves individuals who might hold a germline mutation in specific genes, namely BRCA1 and 2 resulting in several cancer types (specifically colorectal, breast, and ovarian) (Merajver et al., 1995; Bellido et al., 2018). A broad engagement of LOH in cancer development is potentially associated with exposing a physiologically mutated tumor inhibitor gene via deletion of the allele of a wild type (Lynch and De la Chapelle, 2003).

To identify the impact of STR variation in provoking CRC, this study has comprehensively analyzed 16 loci (particularly 15 autosomal STR loci and a separate portion of Amelogenin) for the purpose of investigating possible CRC-stimulated MSI, LOH, loss of manifestation in proteins affected by MMRD and correlations with clinical data on CRC victims.

## Materials and Methods

*Design, Participants and Ethics Statement*

Current research represents a prognostic case-control analysis. The current research had the approval of the Deanship of Scientific Research for Princess Nourah Bint Abdulrahman University. Participants for study and control groups have been selected in cooperation with King Fahad Medical City (KFMC) between August 2020 and November 2020. All methods were carried out in accordance with relevant guidelines and regulations. This study was approved by national regulation committee of ethics, KACST, KSA (study number H-01-R059, IRB LOG number 20-0287). Additionally, the written acquainted permissions have been obtained from the participating patients prior to obtaining their samples.

*Samples Size*

Current research has managed to compile a group consisted of 73 patients from Saudi Arabia. Among participants, 43 patients were diagnosed with CRC, while the rest 30 were healthy and included in the control subgroup. Prior to obtaining specimens, all respondents signed a written consent agreement with no exclusions. Moreover, they managed to fill a special questionnaire to provide data on their personal medical history. The approval from KACST, the ethics board located in Riyadh (KSA), was received after validating the research protocol. 

*Inclusion criteria*

1. Participants were with histologically and invasively approved CRC.

2. Participants unexposed to chemotherapy/radiotherapy previously.

*Exclusion criteria*

1. Participants having other cancer complications.

2. Participants passing through routine neo-adjuvant/adjuvant chemotherapy or radiotherapy. 

Diagnosing of pathologies in clinical setting is presented in Table 1.

*Study Protocol*

*Histopathological Tests*

CRC samples of fixed size (3 x 3 x 2 cm), along with adjoining cancer-free tissues have been obtained from both groups of participants. They were specifically processed with 10% solution of neutral buffered formalin (developed and provided by Sigma-Aldrich, Merck KGaA, Darmstadt, Germany). Processing was organized for eight hours at room temperature conditions. Dehydration of tissues has been completed by increasing grades of alcohol; afterwards, dehydrated tissues were purified in xylene and then integrated into separate paraffin blocks. Almost 3-5µ segments have been sectioned to be included in a typical histopathology trial. One slide was eventually attributed to each paraffin block. Particularly, Eosin and Haematoxylin staining was operated on a slide to cover the full size of a tissue studied. Finally, the stained regions have been screened to get microscopic photographs; for this purpose, a photomicroscope was utilized to estimate the histopathological segments.

*Genetic Analysis*

*Extracting DNA Samples*

Purification of DNA has been completed in relation to blood samples (control group) and CRC samples, along with taking biopsy over the adjoining cancer-free tissues (N-CRC) in the patient group. In general, 20 ml of blood for analysis were obtained; in turn, 30 mg CRC and N-CRC biopsies were surgically obtained as well. Extraction of DNA has been operated with a help of a Qiagen DNA isolation set of instruments (Cat No. 69506, Qiagen, Hilden, developed in Germany). Extraction has been completed in accordance with manufacturer’s manual. 

DNA extracted has been measured by using a NanoDrop spectrophotometer (Model ND-2000-Spectrophotometer UV/Visible). The special wavelength (260 nm) has been applied. Instrumentally, Optical Density (OD) of 1 at 260 nm was attributed to 50 ng/μl of DNA composition. In this sense, the overall DNA concentration could be simply quantified by correlating with OD measurements, which resulted in the following formula:

μg/ml of DNA = A260 X 50 X dilution factor

The principal aim of calculating DNA is to identify the proper quantity of DNA sample to integrate in a PCR amplifying of STR loci to prevent erroneous and exaggerating input and related artifacts. Quantifying the DNA amounts in a specimen is crucial for studies involving PCR methodology, as a limited concentration array is optimally suited for the multiplex STR genotyping procedure.

*DNA Amplification*

PCR can be explained as an enzymatic activity involving multiple replications of the DNA’s certain area to generate several duplicates of a special sequence (Garnett et al., 2012). In a study, selected genetic loci have been amplified by applying the PowerPlex®16 System (developed by Promega Co, United States). This system ensured concurrent amplification and appropriate isolation of 16 STR loci. 

Their decoding names are the following D3S1358, D13S317, D16S539, D18S51, CSF1PO, TH01, vWA, D21S11, D7S820, D5S818, TPOX, D8S1179, FGA, in addition to Penta E, Penta D and Amelogenin (protein isoform).

The list of alleles presented and developed in the allelic ladder, in addition to the genotype secured by the PowerPlex^®^16 System Control DNA 2800M (provided by Thermo Fisher Scientific) have been described in detail in Table 2. Hence, PCR reactions have been organized in compliance with the developer’s instructions using the Gene-Amp^®^ PCR system 9700 thermal cycle (provided by Applied Biosystems). Dilution of specimens has been organized by using deionized water (DI) which was conditional on concentration of DNA. The overall amount of specimens (quantity of samples, positive control samples, negative control samples, as well as potential pipetting errors) have been quantified. Afterwards, PCR amplification procedure has been carried out in an approved quantity of 25μL consisting of 5μL of the PowerPlex® HS 5X Master Mix along with 2.5μL of the PowerPlex®16 HS 10X Primer Pair Mix; it also contained 17.5μL of extracted DNA sample (1ng). Thermal cycler (Applied Biosystems^®^ 2720) has been applied in accordance with the manufacturer’s manual.

*STR Genotyping*

Capillary electrophoresis is viewed as a main procedure of isolating and identifying STR alleles applied in forensic and medical sciences (Ryland et al., 2015). The PCR outputs have been estimated by using capillary electrophoresis in terms of ABI 3130 Genetic Analyzer (provided by Applied Biosystems) to identify STR markers in specimens obtained. Every reaction mix contained 8.6 μl of Hi-Di formamide as well as 0.4 μl of Genescan-500 LIZ Size Standard. Notably, every specimen’s PCR output (in amount of 1.0 μL) showing the ultimate reaction volume of 10μL has been embedded to a genotyping plate equipped with 96 wells and then coated by the special membrane wall. They were later centrifuged to make sure that concentration levels in each well reached the bottom. Afterwards, specimens have been denatured during 3 min at established temperature 95°C to be further quickly chilled towards close-to-the-ice condition (at temperature 4°C) before putting samples into the toolkit. Upon successful chilling, specimens have been placed into the 3130 Genetic Analyzer.

*Genetic Evaluation and Statistical Estimations*

Specimens have been evaluated by utilizing the specialized software, GeneMapper® IDX (version 1.1). STR-related frequencies of the identified alleles have been quantified by implementing GenAlEx (V. 6.503). Statistical calculations have been completed by utilizing Statistical Package for Social Sciences program (version 21.0). Qualitative input has been presented in number/percent format to have a comparative goodness-of-fit analysis (*χ*^2^ testing). Constant variables have been presented as mean ± SD, being further contrasted with the two-sided Student’s T-test. Eventually, a P value > 0.05 has been interpreted as statistically relevant.

## Results

*Histological Findings*

**Figure 1:**
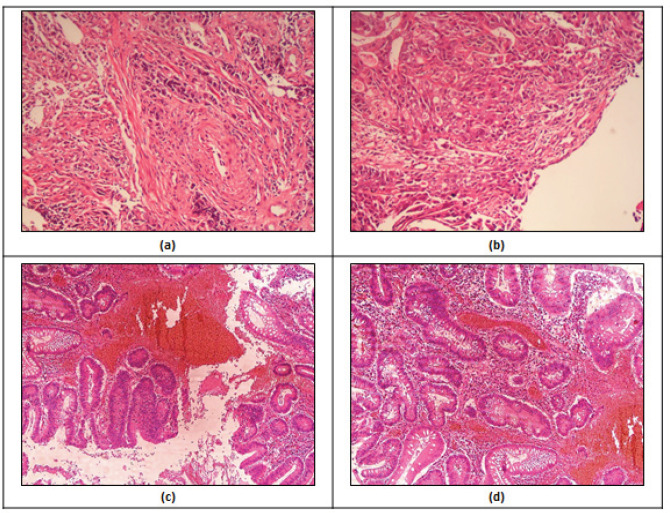
Photomicroscopic Images of: a) H&E-stained CRC histological specimens; and b) stained histological specimens (zoomed at magnitude ×50) of adjoining cancer-free CRC tissues demonstrating the crypts, lamina propria, muscularis mucosae, submucosa as well as lymphoid aggregates within adequate conditions. Moreover, c) and d) stained histopathological specimens (zoomed at magnitude ×100) of MCRA covering the mucous environment and demonstrating an excessive well-identifiable adenocarcinoma; the critical signs are polymorphic epithelium, huge and unstable lumen glands incorporating signs of blood and tumor-related inflammation; moreover, several cells from patients were degraded and incorporated MCRA as well as T5N2M1, showing early signs of developing metastases in liver and abdominal sites

Histological specimens have been taken from CRC patients from two particular areas, namely cancer-affected tissues and adjoining non-affected CRC tissues. Then, the specimen derived have undergone HandE-staining as demonstrated in the Methodology to be further investigated with a help of a light microscope (Figure 1a and b). Photomicroscopic analysis of stained specimens related to adjoining cancer-free tissues revealed the histological structures within the norm, demonstrating proper cellular organization of CRC tissues. In opposition, CRC tissues taken from another area revealed mucinous CRC adenocarcinoma (MCRA) structures associated with increased reproduction disorder within mitotic cells; in addition, distinguished pleomorphism, along with huge and unstable lumen glands incorporating blood and tumor inflammatory signs have been detected (Figure 1 c, and d).

*DNA Concentration*

Table 2 provides a summary on the mean DNA concentrations in samples. A considerable distinction between CRC patients and the control participants on quantities of DNA extracted have been identified with a help of a NanoDrop spectrophotometer having a P-value (P < 0.001) as referred to the source of DNA. Additionally, a remarkable distinction (P < 0.001) in DNA quantities in CR tissues has been detected (cancer-affected and cancer-free tissues). 

**Figure 2:**
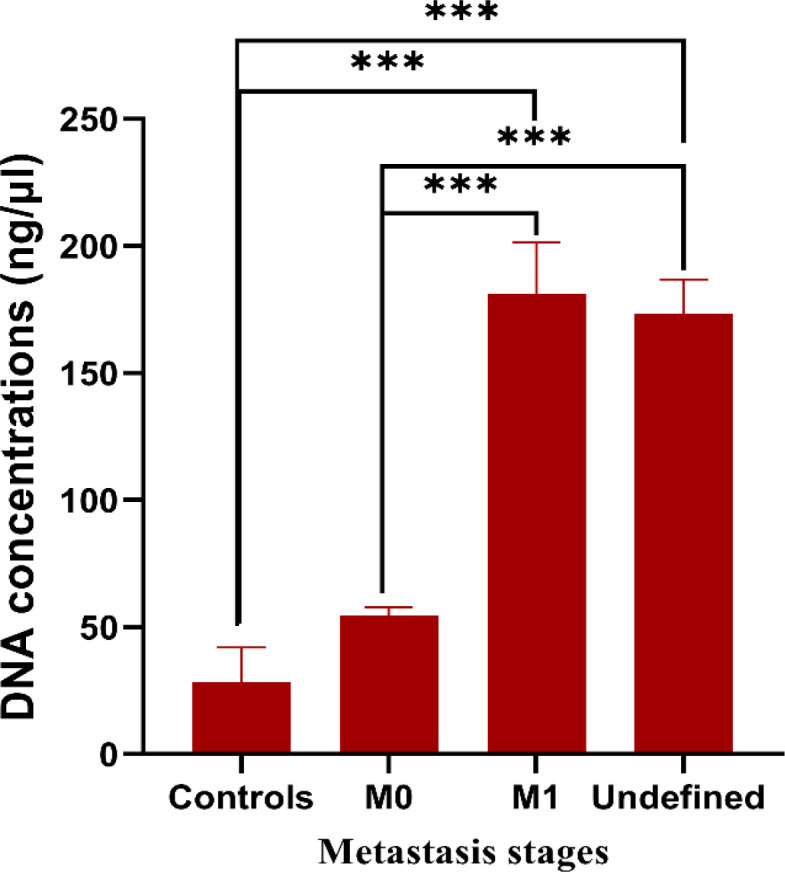
The Relationship between Metastasis Development Phases and DNA Concentrations (ng/μl) Focusing on CR Tissues of CRC Patients. Values derived are presented as (mean ± SEM) passing through a triad of tests for every specimen. P value (***P < 0.001, **P < 0.01, *P < 0.05) has been evaluated for the values of DNA concentrations regarding metastasis-affected participants with CRC (M1 and undetermined metastasis phases) compared to the mean value of the same concentrations participants with CRC (M0) without metastasis signs and in contrast to values from the control group

Furthermore, Figure 2 illustrates the relationship between the metastasis development phases and DNA concentrations (ng/μl) focusing on CRC patients’ cancer tissues. The data indicated that DNA concentrations for participants diagnosed with CRC (M1 and uncertain metastasis development phases) had been dramatically (P < 0.001) higher compared to the ones in CRC (M0) patients without signs of metastasis and the ones from the control group participants. 

*Autosomal STR Allocation*

Of all potential alleles within the loci studied, only 8 appropriate alleles were identified in the locus D13S317 (specifically 7, 9, 10, 11, 12, 13, 14, 15). In turn, 6 alleles were detected in the locus D3S1358 (specifically 12, 13, 14, 15, 16, 17). Additionally, another 7 alleles were detected in the locus D16S539 (specifically 8, 9, 10, 11, 12, 13, 14). Importantly, 15 alleles were identified in the locus Penta E (specifically 6, 7, 8, 9, 10, 11, 12, 13, 14, 15, 16, 17, 18, 19, 20). Penta D revealed 6 appropriate alleles (namely 8, 9, 10, 11, 12, 13). Furthermore, 5 alleles were found in the locus TH01 (namely 7, 8, 9, 10, 11). In the locus vWA, 9 alleles were determined (namely 11, 12, 13, 14, 15, 16, 17, 18, 19). Similarly, 12 alleles were detected in the locus D21S11 (specifically 26, 27, 28, 29, 30, 31, 31.2, 32, 33, 34, 35, 37). The locus D7S820 contained 7 appropriate alleles (such as 6, 7, 8, 9, 10, 11, 12). Another 5 appropriate alleles were found in the locus D5S818 (specifically 7, 8, 10, 12, 13). Another 14 alleles were identified in the locus D18S51 (namely 10, 11, 12, 13, 13.2, 14, 14.2, 15,16, 17, 18, 19, 20, 23). Moreover, another 6 alleles were found in the locus D8S1179 (specifically 9, 10, 11, 12, 13, 14). We managed to identify 5 appropriate alleles in the locus TPOX (namely 6, 7, 8, 10, 11) and 7 alleles in the locus CSF1PO (specifically 8, 9, 10, 11, 12, 13,15). Finally, 16 alleles have been detected in the locus FGA (namely 17, 18, 19, 20, 21, 22, 23, 24, 25, 26, 26.2, 27,28, 29, 30, 31.2). Findings were associated with all study’s participants. 

Table 3 describes the frequencies of two different mutations in 16 STR loci identified in CRC samples with no essential (P≤0.05) distinction in mutation frequencies to the adjoining N- CRC samples. A sum of 1168 loci has been studied overall. It was found that the common frequency of two specific mutations (LOH and MSI) were not greatly different by comparing CRC specimens and N-CRC specimens. Moreover, the genetic deviations have been observed in 5 STRs among other 16, such as Amelogenin, D21S11, D18S51, D8S1179 and CSF1PO. Within these 5 loci examined, 3 specific loci (namely D21S11, D18S51, and CSF1PO) showed considerable (P≤0.05) distinctions between CRC samples and the adjoining N-CRC specimens and in contrast to the control group participants. Moreover, the locus D18S51 has been identified as the most affected one; meanwhile, the locus D8S1179 was categorized as the least affected one with LOH-affected mutation type in DNA of CRC specimens. Additionally, the MSI-affected mutation was identified only in DNA of CRC specimens with no presenting mutations in DNA within the adjoining N-CRC samples. In general, no typical mutations in other loci were identified in relation to the participants from this study. The allocations of mutations identified in 16 STR loci have been broadly presented in Table 4. 

**Figure 3:**
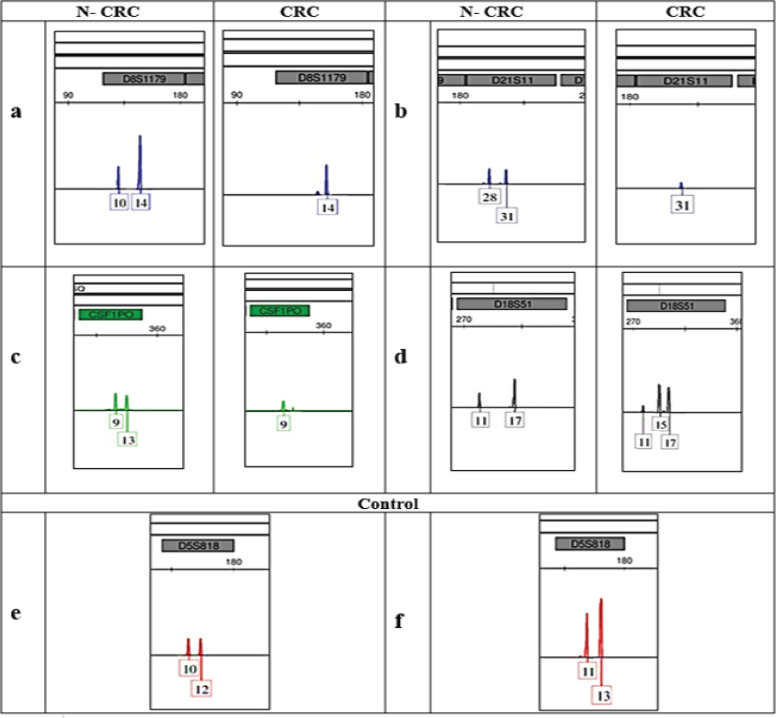
An Electrophoregram of Special Images Demonstrating Genetic Deviations in STR Configurations. LOH was identified in loci such as D8S1179 (a), D21S11 (b), and CSF1PO (c). MSI was detected in the locus D18S51 (d) in CRC samples contrasted to the adjoining N-CRC specimens. Moreover, (e) and (f) revealed the locus D5S818 from two separate specimens taken from the control cohort

Figure 3 provides information on several samples of CRC patients demonstrating the loci affected, such as D21S11, D18S51, D8S1179 and CSF1PO. It also indicates about genetic deviations presented in the alleles as contrasted to specimens from the adjoining N-CRC tissues. Nonetheless, any genetic deviations and mutations were absent in the alleles within the locus D5S818 in specimens of the control group participants. 

**Figure 4:**
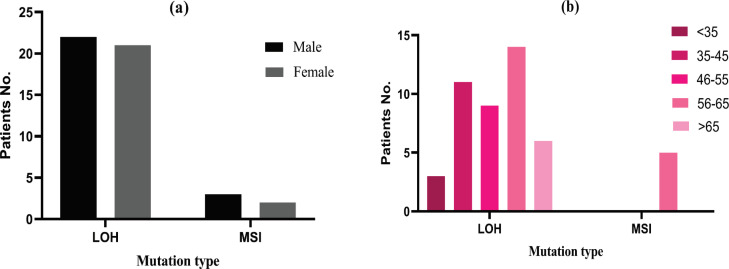
Correlation between Mutation Types (LOH and MSI) in STR Loci and Factors of Gender (a) and Age (b) among CRC Participants (n = 43). Values are presented as (mean ± SEM, n = 43) passing through a triad of tests for every specimen

Figure 4 provides the information on correlations between mutation type within STRs and factors of gender (a) and age (b) among CRC participants. Findings showed no distinctions based on the gender factor for all participants of the research. However, regarding the age factor, there were certain distinctions, demonstrating that majority of participants with CRC incorporating mutations of LOH-type fit the exact age interval (56-65 years). The minority of participants with CRC and mutations of LOH-type was associated with the age interval below 35 years.

Moreover, mutation of MSI-type was categorized as an extra allele inside the same loci affected (namely D21S11, D18S51, D8S1179 and CSF1PO) in samples of 5 CRC patients (among all 43 participants from the group) at specific ages, such as 56, 59, 61, 62 as well as 63 years. Thus, the most participants with CRC affected by mutation of MSI-type were also aged between 56 and 65.

**Table 1:** Study Participants (Patients and Control Groups)

Clinical-pathological Information	Patient Group 43 (%)	Control Group 30 (%)
Gender		
Male	22 (51.16)	14 (46.66)
Female	21 (48.84)	16 (53.33)
Age (years)		
<35	3 (6.97)	12 (40.0)
35-45	11 (25.58)	10 (33.33)
46-55	9 (20.93)	8 (26.66)
56-65	14 (32.56)	0
>65	6 (13.95)	0
Site		
Ascending colon	5 (11.63)	0
Descending colon	3 (6.98)	0
Cecum	8 (18.60)	0
Rectum	10 (23.26)	0
Sigmoid colon	17 (39.53)	0
Total	43 (100)	0
Tumor thickness (mm)		
0-10	8 (18.60)	0
10-20	28 (65.12)	0
>20	7 (16.28)	0
Total	43 (100)	0
Metastasis		
M0	8 (18.60)	0
M1	21 (48.83)	0
Undefined	14 (32.56)	0
Total	43 (100)	0
Nodal status		
N0	0	0
N1	25 (58.14)	0
N2	18 (41.86)	0
Total	43 (100)	0
UICC stage		
Stage 0	0	0
Stage I	0	0
Stage II	8 (18.60)	0
Stage III	24 (55.81)	0
Stage IV	11 (25.85)	0
Total	43 (100)	0

**Table 2:** DNA Concentration (ng/μl) for Study Participants’ Specimens

Participants	DNA concentration (ng/μl)	P
Controls (30)	28.44 ± 18.63	0
Patients (43): colorectal cancer specimens (CRC)	362.53 ± 38.51	
Patients (43): the adjoining non-cancerous tissues (N- CRC)	208.34 ± 29.47	0

Student’s T-test for constant variables, Pearson’s X^2^ test for definitive variables.

**Table 3:** The Correlation between Mutation Types (LOH and MSI) Identified in Autosomal 16 STR loci and Tissues Obtained from CRC Patients (n=43): CRC Specimens and the Adjoining N-CRC Tissues

Mutation type	No. of mutations in CRC samples	Mutation frequency (total no. of loci = 1168)	No. of mutations in N-CRC samples	Mutation frequency (total no. of loci = 1168)	OR (95% CI)	P value
LOH	19	0.0162	7	0.0059	2.74 (0.0129 - 0.0217)	0.617
MSI	5	0.0042	0	0	Undefined	0.667
Total	24	0.0205	4	0.0034	6.02 (0.0097 – 0.0183)	0.465

**Table 4:** Allocation LOH and MSI Mutations Identified in 16 STR Loci in CRC Samples

STR Locus	Chromosome #	Total mutation rate (%)	Number of mutations in CRC		Total mutation rate (%)	Number of mutations in N-CRC		P value
			LOH	MSI		LOH	MSI	
Amelogenin	Sex	2 (0.171)	2	0	1 (0.085)	1	0	0.234 (NS)
D3S1358	3	0 (0.00)	0	0	0 (0.00)	0	0	NA
D13S317	13	0 (0.00)	0	0	0 (0.00)	0	0	NA
Penta E	15	0 (0.00)	0	0	0 (0.00)	0	0	NA
D16S539	16	0 (0.00)	0	0	0 (0.00)	0	0	NA
D18S51	18	9 (0.770)	7	2	2 (0.171)	2	0	0.00
CSF1PO	5	6 (0.513)	5	1	1 (0.085)	1	0	0.00
Penta D	21	0 (0.00)	0	0	0 (0.00)	0	0	NA
TH01	11	0 (0.00)	0	0	0 (0.00)	0	0	NA
vWA	12	0 (0.00)	0	0	0 (0.00)	0	0	NA
D21S11	21	5 (0.428)	4	1	2 (0.171)	2	0	0.009
D7S820	7	0 (0.00)	0	0	0 (0.00)	0	0	NA
D5S818	5	0 (0.00)	0	0	0 (0.00)	0	0	NA
TPOX	2	0 (0.00)	0	0	0 (0.00)	0	0	NA
D8S1179	8	2 (0.171)	1	1	1 (0.085)	1	0	0.234 (NS)
FGA	4	0 (0.00)	0	0	0 (0.00)	0	0	NA

NS, not significant; NA, not applicable

## Discussion

Findings in a recent research literature emphasized on a growing tendency of CRC progression among population of Saudi Arabia (Al-Qahtani et al., 2020; Althubiti and Eldein, 2018; Alyabsi et al., 2020; Chaudhri et al., 2020). Studies have also revealed dramatic disparities of CRC development in relation to factors of gender, age, and geography (Bazarbashi et al., 2017). CRC was found to be the third most prevalent global form of oncology by 2012 (International Agency for Research on Cancer, 2012); moreover, it was categorized as the third most widespread cancer-related diagnosis in many Middle East states, being the second oncology development by popularity in the KSA (Bazarbashi et al., 2017). 

In the past years, STR profiling methods have brought limited innovations, ensuring the traditional yet complicated strategy of capillary electrophoresis (CE) applied to screen multiple loci simultaneously (Gymrek et al., 2012).

Due to increase of international DNA datasets, STR loci used in forensic science improved the discriminatory potential of investigations and enhanced database interoperability. Current STR assays involving CE or MPS (known as massively parallel sequencing) strategies accept the necessity of extending research focus over multiple STR loci. Because of benefits of bigger multiplexing and improved identification of sequence variations, the use of MPS methods for implementing STR evaluation has deeply reinforced discrimination capacities. Therefore, it can further assist in detailed DNA mixture translation by multiplying the amounts of alleles analyzed. Unfortunately, high-performance assays have been scarcely used for managing and expanding forensic DNA databases (Kim et al., 2017). 

Research works focusing on STR variation have been completed in recent years to discover and project the genetic configuration that is subjected to cancer characteristics. The current study involved a cohort of 73 subjects (30 healthy individuals and 43 patients with CRC). They were systematized by gender, age, geography, and histological characteristics – for example, tumor density (mm), metastasis extent, nodal status, and phase of UICC. Characteristics of study’s population indicated that morbidity of CRC among patients was not correlated with patients’ gender, yet correlations with age, in the diapason between 35 and 65, were valid and visible. 

Additionally, histological tests have been completed by using HandE staining technique and a special light microscope. Samples were taken from two locations: CRC tissues and the adjoining N-CRC tissues. Specimens were obtained from CRC-affected patients by keeping a least length of 10 cm between the sections. The outcomes revealed specific histopathological structures in CRC tissues contrasted to the cancer-free histological structures related to the adjoining N-CRC tissues. 

Findings from the current study indicated that DNA concentrations were associated with a considerable growth of parameters of DNA samples from CRC tissues in contrast to N-CRC tissues (P˂0.001) as well as values from the control group of participants (P˂0.001). A vivid relationship between the quantities of DNA examined in CRC participants and the tumor’s metastasis development phases was detected (Lo et al., 2017; Sozzi et al., 2003). Hence, it was found that considerably increased levels of DNA concentration have been present in the tissues of CRC patients with developing metastasis in contrast to metastasis-free tissues and the control group. Findings achieved have been corresponding to reports from previous studies on a similar topic (Li et al., 2020; Perdyan et al., 2020).

In a study completed by Leon et al. and based on radioimmunoassay to identify DNA, a comparison of serum DNA amounts among 173 patients having diverse types of cancer with DNA amounts of 55 healthy participants was completed. Notably, DNA concentrations considerably increased in the cancer-affected cohort in contrast to the control cohort. Additionally, greatly elevated amounts of DNA were discovered in the patients’ serum having metastasis in contrast to metastasis-free participants (Leon et al., 1977). Findings are formally compliant with previous studies informing about increased DNA in the cancer-affected tissues, blood serum or blood plasma in patients with related diagnosis (Leon et al., 1977; Usadel et al., 2002; Wang et al., 2003). 

Aside from increased DNA concentrations, identified alleles in the 16 STR loci have been examined from the specimens obtained from two locations of cancer-affected patients to compare them with DNA profiles of 30 healthy participants from the control group. As noted before, the capillary electrophoresis was used. In terms of quality, frequencies of examined alleles for every STR loci have been comparatively analyzed between the specimens and statistically estimated. The current research also examined the impact of LOH and MSI factors with a purpose of identifying markers associated with cancer. 

In addition, 1168 loci have been examined in sum. The general level of mutation types (namely LOH and MSI) was not found dramatically distinct between CRC and N-CRC samples. More to say, genetic deviations have been recorded in 5 loci (among 16 STR loci), specifically in D21S11, D18S51, D8S1179, CSF1PO and Amelogenin. Among 5 loci detected, 3 loci were found to have increased values (P≤0.05) of dramatic differences in the frequency of selected alleles of CRC samples and N- CRC samples, also compared to specimens from the control group. This included D21S11, D18S51, and CSF1PO loci. Findings from past studies revealed about 3 particular loci – namely D18S51 (Diep et al., 2003; Szych et al., 1999; Vauhkonen et al., 2004), D21S11 (Cho et al., 2014; Vauhkonen et al., 2004), and CSF1PO (Filoglu et al., 2014) – that were attributed to CRC occurrence. This overall corresponds to our individual findings. Hence, it might be proposed that three identified loci can potentially serve as genetic markers concerning CRC diagnosing. MSI was identified only in DNA samples associated with CRC; still, there were no mutations present in DNA of the adjoining N- CRC tissues. Importantly, Alonso and colleagues received the same outcomes upon their examination of CRC, when it was identified that somatic MS repeats were heavily inconsistent (Filoglu et al., 2014). 

In conclusion, concluding all remarks, this study has demonstrated that DNA concentrations in CRC samples dramatically elevated in contrast to N-CRC. Moreover, it was found that mutation rates for LOH and MSI types were not considerably different between DNA specimens from CRC tissues and the adjoining N-CRC tissues. Still, 3 particular loci (namely D21S11, D18S51, and CSF1PO) were identified as having relevant and considerable (P≤0.05) distinctions in frequencies of the allele between CRC specimens and N-CRC samples, especially in comparison with data on the control cohort. It is supposed that 3 identified loci might serve as markers for diagnosing CRC. 

Furthermore, CRC tissue samples might be potentially utilized as a DNA provider for forensic DNA assay. Formally, cancer-affected tissues might manifest MSI and LOH effects and thus compromise interpretation of output, if STRs are used in a forensic practice. This study has a limitation in providing restricted sample sizes. Additionally, it is worth saying that the forensic investigation needs a thorough interpretation of MSI and LOH outcomes along with data from microscopic evaluation of tissue samples. For this reason, future studies should involve a bigger size of samples in working with Saudi patients diagnosed with CRC.

*Abbreviations*

CRC: colorectal cancer 

N-CRC: the adjoining normal (non-cancerous tissues) 

MSI: microsatellite markers 

STRs: short tandem repeats 

MMRD: mismatch repair deficiency 

LOH: loss of heterozygosity 

CNN: copy number neutral 

OD: Optical Density 

DI: deionized water 

PCR: Polymerase chain reaction

CE: capillary electrophoresis 

MPS: massively parallel sequencing

## Author Contribution Statement

Al-Qahtani WS participated in experimental works, investigation, participated in data analysis, and wrote the original draft and submitted the paper as a corresponding author. Al-Hazani TM participated in experimental works, investigation, participated in data analysis, and helped review and editing the draft. Alsafhi FA performed experimental works, investigation, participated in data analysis, and helped review and editing the draft. Alotaibi MA participated in experimental works, investigation, participated in data analysis, and helped review and editing the draft. Domiaty DM participated in experimental works, investigation, participated in data analysis, and helped review and editing the draft. Al-Shamrani SM participated in experimental works, investigation, participated in data analysis, and helped review and editing the draft. Alshehri E participated in experimental works, investigation, participated in data analysis, and helped review and editing the draft. Alotaibi AM participated in experimental works, investigation, participated in data analysis, and helped review and editing the draft. Alkahtani S participated in investigation, participated in data analysis, and helped review and editing. The draft All authors read and approved the final manuscript.

## References

[B1] Al-Qahtani WS, Al-Olayan E, Albani FG (2020). Utility of KRAS gene and clinicopathological features in the assessment of the risk of type 2 diabetes in the etiology of colon cancer. J Med Genet.

[B2] Althubiti MA, Eldein MM (2018). Trends in the incidence and mortality of cancer in Saudi Arabia. Saudi Med J.

[B3] Alyabsi M, Alhumaid A, Allah-Bakhsh H, Alkelya M, Aziz MA (2020). Colorectal cancer in Saudi Arabia as the proof-of-principle model for implementing strategies of predictive, preventive, and personalized medicine in healthcare. J EPMA.

[B4] Bazarbashi S, Al Eid H, Minguet J (2017). Cancer incidence in Saudi Arabia: 2012 data from the Saudi cancer registry. Asian Pac J Cancer Prev.

[B5] Bellido F, Sowada N, Mur P (2018). Association between germline mutations in BRF1, a subunit of the RNA polymerase III transcription complex, and hereditary colorectal cancer. J Gastroenterol.

[B6] Chaudhri E, Fathi W, Hussain F, Hashmi SK (2020). The increasing trends in cases of the most common cancers in Saudi Arabia. JEGH.

[B7] Chen H, Xu L, Yin L (2014). i TRAQ-based proteomic analysis of dioscin on human HCT-116 colon cancer cells. J Proteom.

[B8] Cho YB, Hong HK, Choi YL (2014). Colorectal cancer patient–derived xenografted tumors maintain characteristic features of the original tumors. J Surg Res.

[B9] Dang Z, Li L, Kong X (2020). Evaluation of allelic alterations in short tandem repeats in papillary thyroid cancer. Mol Genet Genomic Med.

[B10] Diep CB, Thorstensen L, Meling GI (2003). Genetic tumor markers with prognostic impact in Dukes’ stages B and C colorectal cancer patients. J Clin Oncol.

[B11] Filoglu G, Bulbul O, Rayimoglu G (2014). Evaluation of reliability on STR typing at leukemic patients used for forensic purposes. Mol Biol Rep.

[B12] Garnett MJ, Edelman EJ, Heidorn SJ (2012). Systematic identification of genomic markers of drug sensitivity in cancer cells. Nature.

[B13] Gymrek M, Golan D, Rosset S, Erlich Y (2012). lobSTR: a short tandem repeat profiler for personal genomes. Genome Res.

[B14] Kim EH, Lee HY, Kwon SY (2017). Sequence-based diversity of 23 autosomal STR loci in Koreans investigated using an in-house massively parallel sequencing panel. Forensic Sci Int Genet.

[B15] Leon SA, Shapiro B, Sklaroff DM, Yaros MJ (1977). Free DNA in the serum of cancer patients and the effect of therapy. Cancer Res.

[B16] Li QJ, Mao YP, Guo R (2020). A nomogram based on serum biomarkers and clinical characteristics to predict survival in patients with non-metastatic nasopharyngeal carcinoma. Front Oncol.

[B17] Lichtenstein P, Holm NV, Verkasalo PK (2000). Environmental and heritable factors in the causation of cancer—analyses of cohorts of twins from Sweden, Denmark, and Finland. NEJM.

[B19] Lynch HT, De la Chapelle A (2003). Hereditary colorectal cancer. NEJM.

[B20] Merajver SD, Frank TS, Xu J (1995). Germline BRCA1 mutations and loss of the wild-type allele in tumors from families with early onset breast and ovarian cancer. Clin Cancer Res.

[B21] Perdyan A, Spychalski P, Kacperczyk J, Rostkowska O, Kobiela J (2020). Circulating Tumor DNA in KRAS positive colorectal cancer patients as a prognostic factor–a systematic review and meta-analysis. Crit Rev Oncol Hemat.

[B22] Ryland GL, Doyle MA, Goode D (2015). Loss of heterozygosity: what is it good for?. J Med Genet.

[B23] Sozzi G, Conte D, Leon M (2003). Quantification of free circulating DNA as a diagnostic marker in lung cancer. J Clin Oncol.

[B24] Szych C, Staebler A, Connolly DC (1999). Molecular genetic evidence supporting the clonality and appendiceal origin of pseudomyxoma peritonei in women. Am J Pathol.

[B25] Usadel H, Brabender J, Danenberg KD (2002). Quantitative adenomatous polyposis coli promoter methylation analysis in tumor tissue, serum, and plasma DNA of patients with lung cancer. Cancer Res.

[B26] Vauhkonen H, Hedman M, Vauhkonen M (2004). Evaluation of gastrointestinal cancer tissues as a source of genetic information for forensic investigations by using STRs. Forensic Sci Int.

[B27] Wang BG, Huang HY, Chen YC (2003). Increased plasma DNA integrity in cancer patients. Cancer Res.

